# The Brazilian Version of the Mystical Orientation Scale Revised (MOSR-BR): An Exploratory Study

**DOI:** 10.1007/s10943-026-02624-3

**Published:** 2026-03-31

**Authors:** Ana Cláudia Mesquita Garcia, Everson Meireles, Eliza Mara das Chagas Paiva, Lucas Oliveira Maia, Luís Fernando Tófoli, Leslie J. Francis

**Affiliations:** 1 Interdisciplinary Center for Studies in Palliative Care, School of Nursing, Federal University of Alfenas, Alfenas, Brazil; 2 Laboratory for Interdisciplinary Studies on Psychoactive Substances (LEIPSI) , State University of Campinas, Campinas, Brazil; 3 Health Sciences Center, Federal University of Recôncavo da Bahia, Santo Antônio de Jesus, BA Brazil; 4 Brain Institute, Federal University of Rio Grande Do Norte, Natal, Brazil; 5 Centre for Research in Intellectual and Developmental Disabilities (CIDD), University of Warwick, Coventry, UK; 6 World Religions and Education Research Unit, Bishop Grosseteste University, Lincoln, UK

**Keywords:** Mystical orientation, Death transcendence, Spiritual wellbeing, Psychedelic substances, Brazil

## Abstract

Mystical experiences are commonly described in the context of psychedelic use and other forms of expanded states of consciousness. Although the Mystical Orientation Scale Revised (MOSR) has been used in international studies, no Brazilian Portuguese version had been tested to date. This exploratory study aimed to translate, culturally adapt, and examine the psychometric properties of the MOSR for the Brazilian population (MOSR-BR). The process followed international guidelines, including translation, back-translation, expert committee review, and pre-testing. The final version was applied to a sample of 505 Brazilian adults. Exploratory factor analysis supported a unidimensional structure, explaining 47.6% of the variance. The MOSR-BR showed high internal consistency (α = .95), slightly higher than the original and Italian versions. Construct validity was evidenced by significant positive correlations between MOSR-BR and all subscales of the Death Transcendence Scale, as well as with the Religious and Existential Well-Being subscales of the Spiritual Well-Being Scale. The scale also discriminated between psychedelic substance users and non-users, with higher mystical orientation scores among occasional and frequent users. This exploratory study provides preliminary evidence supporting the value of further research on the MOSR-BR to address acknowledged limitations with the pilot study, including the cross-sectional design, non-causal inferences, recruitment method and participant self-selection, limited generalizability, absence of confirmatory factor analysis and test–retest reliability, and the need for larger and more diverse samples with longitudinal reliability assessment.

## Introduction

From an etymological perspective, the English term mystical derives from a Latin transliteration of the Greek word *musterion*, which is related to *mustes*—a term used to denote “closed mouth,” originating from the verb *muo*, meaning “I shut my mouth.” As a noun, *musterion* came to refer to an initiate in religious mystery cults, in which secrecy and discretion were often required (Partridge, 1958, p. 424). Mystical experience can be defined as a sense of union or identity with something other than oneself (Francis & Louden, 2000). Mysticism lies at the heart of religions, insofar as religion implies belief in a transcendent reality with which it is, in some way, possible to communicate through direct experience (Francis & Louden, 2000).

According to Yaden and Newberg (2022), mystical experience refers to internal states that involve a deeply felt weakening of one’s sense of identity and/or feelings of greater connectedness, up to and including the sensation of complete unity. These feelings extend far beyond the normal sense of self-awareness experienced in daily life (Yaden & Newberg, 2022). In ordinary consciousness, there is typically the impression that, on one side, there is a self, and on the other, the external world. However, in certain circumstances, this sense of identity can be reversed—during such moments, the sense of self may recede into the background and become part of everything else, rather than something separate (Yaden & Newberg, 2022).

Those who undergo a mystical experience often find it difficult to describe. For instance, they may say that, although their awareness of a Presence is more real to them than anything else they know, they remain unable to express it—except through symbols, images, and approximations—and even then, they do not know it in the same way they know other things (Happold, 1963). This is the paradox at the heart of the mystical experience (Francis & Louden, 2000). Hence the enduring difficulty experienced by mystics in conveying their experiences in a language that is easily comprehensible to those who have not undergone such experiences (Francis & Louden, 2000).

In the field of the psychology of religion, the measurement of mystical orientation has been recognized as a valuable approach to investigating individual differences in openness to this type of experience. With the aim of operationalizing this construct, Francis and Louden (2000) developed the Mystical Orientation Scale (MOS), based on the classical typology of mystical experience proposed by Happold (1963). This typology includes seven core characteristics—ineffability, noesis, transiency, passivity, consciousness of the oneness of everything, sense of timelessness, and true ego—which served as the theoretical foundation for the construction of the scale. The MOS proposes three indicators of each of these seven characteristics in order to construct a 21-item measure.

Originally developed for use among Roman Catholic priests, the MOS consists of items that assess the importance attributed to mystical experiences within the context of faith. Psychometric evaluations of the original version demonstrated a clear unidimensional structure and high internal consistency, indicating that the scale effectively measures a coherent psychological disposition toward mystical experience (Francis & Louden, 2000). Although the original MOS was developed in a religious context and was initially designed to reflect an explicitly Christian interpretation of mystical experience, its core elements resonate with broader forms of spirituality (Giordan et al., 2018). As such, later revisions of the scale led to an instrument that operationalizes the concept without specific reference to Christian language. The Mystical Orientation Scale Revised (MOSR) emerged as a promising alternative for exploring mystical orientation beyond institutional religious contexts, enabling its application across increasingly diverse populations and cultural settings (Giordan et al., 2018).

Despite its theoretical relevance and previous use in international studies, until now, no version of the MOSR had been translated, adapted, and validated for the Brazilian population. Considering the observations by Garcia et al. (2025a) regarding the limitation of instruments that assess spirituality-related constructs solely through a religious lens, it is essential to have tools that also encompass forms of spirituality detached from traditional religious beliefs or practices. This need becomes even more evident in research contexts involving non-religious but highly spiritualized groups—such as users of psychedelic substances, for instance—who frequently report experiences of transcendence and existential meaning. In this regard, validating the MOSR for the Brazilian context represents a significant methodological advance, offering a tool capable of assessing mystical orientation in a broader and more inclusive manner, sensitive to contemporary expressions of spirituality that go beyond institutional religiosity.

Therefore, this study aimed to address this gap through the translation, cultural adaptation, and psychometric evaluation of the Brazilian version of the MOSR (MOSR-BR). Through this effort, we seek to provide a reliable and culturally sensitive instrument that can support future research in areas such as spirituality, mental health, and altered states of consciousness.

## Methods

### Procedure

This is a survey-type, cross-sectional study conducted using online data collection, reported according to the STrengthening the Reporting of OBservational studies in Epidemiology (STROBE) guidelines (von Elm et al., 2008). Recruitment and data collection took place from April to June 2022. This study is part of a larger project that aimed to assess spiritual well-being (Garcia et al., 2025a), death transcendence (Garcia et al., 2023), death anxiety (Garcia et al., 2025b), and mystical orientation among users and non-users of psychedelics.

This study assessed validity evidence based on the internal structure, obtained through factor analysis (Rios & Wells, 2014). Thus, the sample size was established according to the recommendation of 10 individuals per instrument item (Terwee et al., 2007). The established sample consisted of at least 250 participants, consisting of adult Brazilian individuals, regardless of gender and health status. Responses from participants who did not complete the study instruments in full were excluded.

A snowball sampling strategy was used to recruit participants. The study was initially disclosed within the researchers’ networks, mainly through messaging apps (Whatsapp, Telegram) and social media (Instagram, Twitter, Facebook). In addition to participating in the study, potential participants were also invited to disseminate the study among their peers. Potential participants received a message containing information regarding the study, a link to the online form, and instructions on how to complete the study’s instruments and provide consent. They were taken to a website with a message thanking them for considering participating in the study if they declined to provide their consent to participate. The Google Forms platform was used to create the online form.

### Measures

#### Sample Characterization and Psychedelic Use

The participants completed an initial set of questions about their age, gender, level of education, professional profile, and perspectives on death/finitude. Regarding psychedelics, the questions asked whether they used these substances in general, with the following response options: “never,” “almost never,” “occasionally,” and “frequently”. Regarding the specific use of substances, the questions asked about: Lysergic acid diethylamide (LSD), ayahuasca, N,N-dimethyltryptamine (DMT), psilocybin, mescaline, ibogaine, cannabis, 3,4-methylenedioxymethamphetamine (MDMA), and ketamine, with the following response options: “I’ve never used,” “I’ve previously used in my life,” “I’ve used it in the last 12 months,” and “I’ve used it in the last 30 days.”

#### Mystical Orientation

Mystical orientation was assessed using the revised version of the Francis and Louden (2000) Mystical Orientation Scale (MOS). The original version of the scale was developed within an explicitly religious context, aimed at measuring mystical experiences among Roman Catholic priests (Francis & Louden, 2000). The scale developed to assess mystical orientation consists of 21 items that inquire about the importance attributed to these experiences in the respondents’ faith life. Regarding its psychometric properties, the scale demonstrated high internal consistency (*α* = .94, indicating that the items reliably measured a coherent construct (Francis & Louden, 2000). Factor analysis supported the unidimensionality of the scale, with the first factor explaining 45.1% of the variance, in contrast to 7.3% explained by the second factor, indicating that the items predominantly assess a single dimension related to mystical orientation (Francis & Louden, 2000). The factorial structure of the 21 items proved to be clear and coherent with the theoretical model of mysticism proposed by Happold (1963). Furthermore, the scale was found to be independent of personality dimensions such as neuroticism and psychoticism, but showed a positive correlation with extraversion, suggesting that mystical experiences are more frequently reported by extraverted individuals (Francis & Louden, 2000). Its items reflect a language centered on religious experience and institutional faith. In contrast, the revised version of the scale (MOSR; Giordan et al., 2018) presents a broader formulation, allowing for the assessment of the importance attributed to mystical experiences regardless of religious affiliation. This reformulation broadens the scale’s applicability to wider spiritual contexts, including non-religious experiences or spiritual states associated with expanded states of consciousness. In respect of the Brazilian version the back-translated versions of the 21-item MOSR were sent to the first author of the original scale’s publication article (Francis & Louden, 2000), who approved them, ensuring their conceptual equivalence (Leslie J. Francis, personal communication, March 5, 2022). MOSR-BR is presented as Appendix A.

#### Death Anxiety

The Death Anxiety Scale (DAS) was originally developed by Templer (1970) on a sample of North American university students. It is a unidimensional instrument composed of 15 items. The original version of the DAS presented internal consistency coefficients equal to .76 (Templer, 1970). The Brazilian version of the DAS (*α* = .77) was developed by Donovan (1993) on a sample of Brazilian adults. The instrument was answered using a numerical scale ranging from 1 to 7. Higher scores indicate higher levels of death-related anxiety (Templer, 1970).

#### Death Transcendence

The Death Transcendence Scale (DTS) was developed by Vandecreek and Nye (1993) on a sample of North American adults. The scale aims to assess the transcendence of death through five modes, namely: religious, mystical, creative, biosocial, and nature. The Brazilian version of the DTS (21-item) used in this study was validated by Garcia et al. (2023), on a sample of Brazilian adults, in the same sample as this study. The internal consistency coefficients of the Brazilian version of the DTS (α) ranged from .94 to .63 (for this study we did not use the *nature* factor of the Brazilian version of the DTS, considering that its *α* = .44) (Garcia et al., 2023). Each response is rated on a Likert scale, ranging from 1 (strongly disagree) to 4 (strongly agree). The negative items’ scores should be inverted regarding the total score. The scores for the five subscales are then calculated by adding the item scores together; these scores can be expressed as the sum or the mean. According to Gjolaj and MacDonald (2011), the responses’ level of investment is indicated by their scores on each subscale.

#### Spiritual Well-Being

Spiritual well-being is understood as a sense of well-being that is experienced when one finds a purpose that justifies commitment to something in life, and that purpose involves an ultimate meaning to life (Ellison, 1983). The Spiritual Well-Being Scale (SWBS) was developed by Paloutzian and Ellison (1991), who based it on the study by Moberg and Brusek (1978), which pointed to a vertical and a horizontal dimension for spiritual well-being. These dimensions became the two factors measured by the scale: religious well-being (RWB), a vertical dimension related to satisfaction in the personal connection with God or with something considered to be absolute; and existential well-being (EWB), a horizontal dimension that refers to the person’s perception of the purpose of life independent of a religious reference (Marques et al., 2009). The Brazilian version of the SWBS used in this study was validated by Garcia et al. (2025a) among Brazilian adults, in the same sample as this study. The Brazilian version contains 18 items divided into two factors: RWB (*α* = .94) and EWB (*α* = .83) (Garcia et al., 2025a). Responses are provided using a six-option Likert-type scale: “Strongly Agree” to “Strongly Disagree.” To obtain the score of the factors, it is necessary to sum the answers of the items that compose them (Marques et al., 2009).

### Participants

The sample comprised 517 people (response rate = 98.3%), of whom 70% were female, whose ages ranged from 19 to 76, with a mean of 38.8 years (*SD* = 12.0). Table 1 includes further information about the participants’ characteristics.

**Table 1:** Sample characterization

Variables / Groups	N	%
*Gender*		
Female	330	63.8
Male	182	35.2
Other	5	1.0
*Marital status*		
Single/no steady partner/widowed	260	50.3
Married/stable union	257	49.7
*Education*		
Up to High School	30	5.8
Undergraduate (complete or incomplete)	204	39.4
Postgraduate	283	54.8
*Religion*		
I have no religion, but I consider myself spiritualized	242	46.8
I have no religion and do not consider myself spiritualized	61	12.0
Catholic	70	13.5
Evangelical/Protestant	13	2.5
Spiritualist	48	9.0
Buddhist	6	1.2
Afro-Brazilian religions	24	5.0
Other*	53	10.0
*Do you have any illness that you consider serious?*		
Yes	41	8.0
No	476	92.0
*Does anyone close to you, whom you consider an important person in your life, have any illness that you consider serious?*		
Yes	197	38.0
No	320	62.0
*Have you ever experienced the loss of someone important (death) in your life?*		
Yes	450	87.0
No	67	13.0
*Do you often talk about death/finitude?*		
Yes	433	83.7
No	84	16.3
Use of psychedelics**		
Never	175	34.0
Almost never	56	11.0
Occasionally	112	21.5
Frequently	174	33.5

(N = 517). *Ayahuasca religions and groups such as *Santo Daime* and ayahuasca (neo)shamanism. **LSD, ayahuasca, DMT, psilocybin-containing mushrooms, ibogaine, cannabis, MDMA, among others

### Analysis

The dimensionality of the MOSR-BR was assessed through Exploratory Factor Analyses, using the Principal Axis Factoring (PAF) extraction method and suppressing factor loadings below .40 (Rios & Wells, 2014). The number of factors to be extracted was determined based on parallel analyses (Horn, 1965). Internal consistency was evaluated using Cronbach’s alpha coefficient, with values above .60 considered acceptable for exploratory studies (Hair et al., 2010). Scores were calculated as the arithmetic mean of item responses, ranging from 1 to 5—higher scores indicate greater mystical orientation.

Pearson’s correlation coefficients were used to examine the pattern of correlation between the mystical orientation measure (MOSR-BR) and other theoretically related constructs (validity based on relationships with external measures). In this case, we expect to find significant correlations, even if of weak magnitude, considering that we are evaluating measures related to the construct of interest (mystical orientation) that are relevant to its understanding. Negative correlations were expected with the DAS (Garcia et al., 2025b), while positive correlations were anticipated with the SWBS and DTS. Positive correlations are expected between the MOSR-BR and both the SWBS and DTS, as mystical orientation reflects a propensity for transcendent experiences that foster spiritual meaning, existential purpose, and a broader interpretation of life and death. To interpret the strength of the relationship, correlation coefficients between .1 and .3 were considered weak, between .4 and .6 moderate, and between .7 and .9 strong (Akoglu, 2018). Significant differences in Mystical Orientation scores between participant groups were also investigated: by participant gender using Student’s t-test, and by patterns of psychoactive substance use using ANOVA. All analyses were conducted at a significance level of *p* ≤ .05.

## Results

### Factor Structure and Internal Consistency of the MOSR-BR

**Fig. 1:**
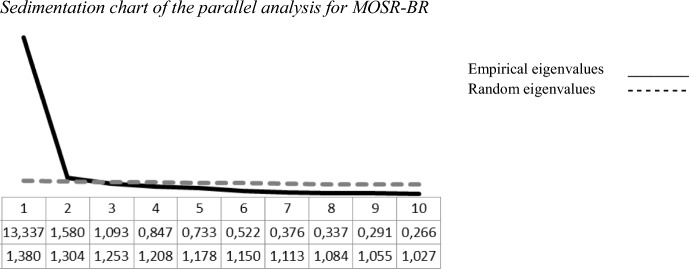
Sedimentation chart of the parallel analysis for MOSR-BR. N = 517

Parallel analysis (Horn, 1965) supported the extraction of one or, at most, two factors, as random eigenvalues surpassed empirical ones from the third factor onward (see Fig. 1).

In the initial factor analysis, the sampling adequacy index was obtained–Kaiser-Meyer-Olkin (KMO) measure = .94 and correlation matrix determinant different from zero, indicating the possibility of extracting factors from the data matrix (Hair et al., 2010). Two factor solutions were tested: a) two oblique factors, which explained 54.3% of the variance, producing items with cross-loadings on both factors and few items grouped in factor 2; and b) a unifactorial solution, which explained 47.6% of the variance, proved to be more parsimonious, interpretable and aligned with the unidimensional treatment given to the scale in previous studies (Francis & Louden, 2000; Giordan et al., 2018), as shown in Table 2.

**Table 2:** Results of exploratory factor analysis and internal consistency analysis of MOSR-BR

Items	Factor	*r*
13. Feeling at one with the universe	.82	.79
21. Sensing the unity in all things	.79	.76
14. Being absorbed within a greater being	.79	.79
15. Sensing the merging of past, present and future	.76	.75
19. Feeling my everyday self absorbed in the depths of being	.76	.73
16. Feeling at one with all living beings	.75	.73
2. Feeling moved by a power beyond description	.74	.71
18. Being conscious only of timelessness and eternity	.73	.71
3. Being aware of more than I could ever describe	.73	.70
17. Losing my everyday self in a greater being	.72	.72
12. Being grasped by a power beyond my control	.69	.70
5. Knowing I was surrounded by a presence	.68	.66
11. Being in a state of mystery outside my body	.67	.67
6. Hearing an inner voice speak to me	.65	.63
7. Seeing brief glimpses into the heart of things	.64	.60
1. Experiencing something I could not put into words	.62	.60
4. Sensing meaning in the beauty of nature	.59	.56
20. Losing a sense of time, place and person	.58	.58
8. Having flashes of insight into what really matters	.57	.54
9. Experiencing passing moments of deep insight	.57	.54
10. Being overwhelmed by a sense of wonder	.54	.51
Item count, 21		
% explained variance, 47.6		
Cronbach’s alpha, .95		

N = 517. *r* = correlation between the item and the sum of the other 20 items. Factor = loadings on principal axis factorization. One-dimensional model converged on three interactions

Descriptive statistics showed a mean mystical orientation score of 4.2 (95% CI: 4.02–4.16), with a standard deviation of .86 and a median of 4.3.

### Construct Validity Based on Relationships With External Measures

Pearson’s correlation coefficients were used to examine the pattern of associations between the MOSR-BR and other theoretically related constructs (see Table 3). Mystical orientation showed moderate positive correlations with the mystical (*r* = .50, *p* ≤ .001) and religious (*r* = .46, *p* ≤ .001) subscales of the DTS. Significant positive correlations (weak) were also observed with the nature (*r* = .23, *p* ≤ .001), creative (*r* = .19, *p* ≤ .001), and biosocial (*r* = .10, *p* ≤ .05) dimensions of the DTS. Regarding the SWBS, mystical orientation correlated positively with both religious well-being (*r* = .31, *p* ≤ .001) and existential well-being (*r* = .15, *p* ≤ .001). A negative, although non-significant, correlation was observed between the MOSR-BR and the DAS.

**Table 3:** Correlations between Brazilian version of the Mystical orientation scale revised and Measures of death transcendence, Spiritual well-being, and Death anxiety

	Mystical orientation
*Death Transcendence Scale*	
Mystical	.50^***^
Nature	.23^***^
Religious	.46^***^
Biosocial	.10^*^
Creative	.19^***^
*Spiritual Well-Being Scale*	
Religious well-being	.31^***^
Existential well-being	.15^***^
*Death Anxiety Scale*	-.07

^*^p < .05, ^***^p < .001. N = 517

### Comparison Between Groups

Group comparisons using ANOVA revealed significant differences in mystical orientation scores across groups based on psychoactive substance use [F(3, 513) = 28.566, *p* ≤ .001]. Post hoc Tukey tests indicated that occasional (M = 4.3) and frequent users (M = 4.4) of substances such as LSD, ayahuasca, DMT, psilocybin, mescaline, cannabis, MDMA, or ketamine scored significantly higher than individuals who had never used (M = 3.7) or rarely used (M = 3.9). Ibogaine users did not show distinctive differences. No significant sex differences were found [t(510) = − 0.551, *p* = 0.582], with similar scores for male (M = 4.0) and female (M = 4.1) participants.

## Discussion

Parallel analyses indicated the extraction of up to two factors for the MOSR-BR; however, the unidimensional solution showed the best fit for the Brazilian version of the scale, explaining 47.6% of the variance. In the study by Giordan et al. (2018) on the Italian version of the MOSR, the scale also demonstrated a unifactorial solution, with the first principal component accounting for 31% of the variance, and all individual items loading on this factor with values ranging from .38 to .67, indicating a robust unidimensional structure. These findings are consistent with those reported by Francis and Louden (2000) in the development study of the MOS, in which the authors defined mystical orientation as a unidimensional construct. They supported the unidimensionality of the MOS based on factor analysis, which revealed the predominance of a single underlying factor responsible for 45.1% of the variance (Francis & Louden, 2000). Although a two-factor structure was also identified, the second factor contributed only marginally to the total variance—just 7.3% (Francis & Louden, 2000). These results suggest that, despite some complexity in the structure of mystical orientation, these versions of the scale, including the Brazilian one, are best represented by a unifactorial solution.

Regarding reliability, the results obtained with the MOSR-BR demonstrated satisfactory internal consistency, with a Cronbach’s alpha coefficient of .95. This value is slightly higher than that reported for both the original version of the scale (*α* = .94) by Francis and Louden (2000) and the Italian version (*α* = .89) by Giordan et al. (2018), suggesting that the items of the MOSR-BR exhibit high homogeneity in measuring the construct. These results reinforce the psychometric robustness of the scale and indicate its suitability for use in different cultural contexts, maintaining stability in the assessment of mystical orientation.

Regarding findings on validity based on relationships with external measures, the MOSR-BR showed positive correlations with all DTS factors (mystical, religious, nature, creative, and biosocial). Mystical orientation, as defined by Francis and Louden (2000), refers to a stable disposition to believe in and remain open to mystical experience. Such a disposition implies a continuous and enduring receptivity to transcendent experiences, which makes its association with the DTS factors plausible. The mystical factor of the DTS describes a state of experience in which individuals perceive themselves as existing beyond time, space, and bodily death, often accompanied by a sense of euphoria and the perception of immortality (Gjolaj & MacDonald, 2011). The religious factor, in turn, relates to the idea of continuity of life after death through the survival of the soul, as taught by various religious doctrines—in other words, a transition from one form of existence to a higher one (Gjolaj & MacDonald, 2011). The correlation identified between the MOSR-BR and the remaining DTS factors (nature, creative, and biosocial) indicates an association between mystical orientation and forms of death transcendence not necessarily linked to religious spirituality. This finding appears to support the purpose of the revised version of the MOS, which seeks to broaden its applicability to wider spiritual contexts beyond formal religious traditions.

The MOSR-BR also showed a positive correlation with the RWB and EWB factors of the SWBS. This association is theoretically consistent, as religious well-being refers to the perceived quality of an individual’s relationship with God or with a reality regarded as absolute, whereas existential well-being concerns a sense of purpose and life satisfaction, regardless of religious affiliation (Marques et al., 2009). Considering mystical orientation as a stable disposition to believe in and remain open to mystical experience (Francis & Louden, 2000), it is expected that individuals with higher mystical orientation would also exhibit higher levels of spiritual well-being—both in its religious and existential dimensions. The positive correlation with these factors suggests that mystical experiences perceived as meaningful may contribute to strengthening both the connection with the sacred and the existential meaning attributed to life, thus reinforcing the convergent validity of the MOSR-BR.

Group comparisons revealed significant differences in mystical orientation scores based on psychoactive substance use: occasional (M = 4.35) and frequent (M = 4.39) users of substances such as LSD, ayahuasca, DMT, psilocybin, mescaline, cannabis, MDMA, or ketamine had significantly higher scores than individuals who had never used (M = 3.67) or rarely used (M = 3.97) such substances. The ability of the MOSR-BR to discriminate between users and non-users of psychedelic substances represents a relevant finding, as the literature indicates that mystical experiences are a central component of psychedelic-induced states. This discriminative capacity of the MOSR-BR is particularly relevant in light of empirical evidence suggesting that mystical experience may be a key psychological mechanism underlying the therapeutic effects of psychedelics.

Walter Pahnke (1969), one of the pioneers in the study of mystical experiences induced by psychedelics, proposed that the psychological phenomena characterizing such experiences can be categorized into nine interrelated dimensions: Unity, Transcendence of time and space, Deeply felt positive mood, Sense of sacredness, The noetic quality, Paradoxicality, Alleged ineffability, Transiency, and Persisting positive changes in attitude and behavior. These experiences—often marked by ego dissolution, a sense of universal interconnectedness, and the absence of self-boundaries, described as oceanic boundlessness—have been associated with symptom reduction and improved quality of life across various clinical contexts (Ko et al., 2022).

In a review study, Ko et al. (2022) analyzed twelve investigations of psychedelic-assisted therapies using psilocybin, ayahuasca, or ketamine to determine the association between mystical experience and symptom reduction in diverse areas, including cancer-related distress, substance use disorders, and depressive disorders, including treatment-resistant cases. Ten of the twelve studies included in the review established a significant association—through correlation, mediation, and/or prediction—between mystical experience and symptom reduction (Ko et al., 2022). These findings underscore the importance of instruments capable of measuring mystical orientation, such as the MOSR-BR, particularly in research and clinical contexts involving altered states of consciousness.

## Limitations

This exploratory study has limitations that fail to meet the exacting criteria specified by Koenig and Al Zaben (2021). First, the use of an online convenience sample limits the generalizability of the findings, as it may not accurately represent the broader Brazilian population. Participants were primarily recruited through digital platforms, which may have resulted in the overrepresentation of individuals with higher education and greater familiarity with spiritual or psychedelic-related themes. Second, the cross-sectional design does not allow for causal inferences regarding the relationships observed between mystical orientation and other variables, such as psychedelic use or spiritual well-being. Another limitation of this study is that confirmatory factor analysis (CFA) was not conducted in an independent subsample. As this represents the first validation of the MOSR in the Brazilian context, an exploratory approach was prioritized, and splitting the sample would have yielded subsamples of limited size for stable parameter estimation. Future research with larger and independent samples should examine the factor structure using confirmatory methods. A further limitation is that the study was not designed to assess test–retest reliability.

## Conclusion

In this exploratory study the Brazilian version of the Mystical Orientation Scale Revised (MOSR-BR) demonstrated satisfactory psychometric properties, supporting its use as a valid and reliable instrument for assessing mystical orientation in Brazilian adults. Its moderate correlations with measures of death transcendence and spiritual well-being, as well as its ability to distinguish between psychedelic users and non-users, reinforce its validity based on relationships with external measures and potential utility in both clinical and research settings. The MOSR-BR offers a culturally adapted tool that broadens the scope of investigation into mystical experiences beyond explicitly religious frameworks, contributing to the growing field of studies on spirituality and health. On this basis the exploratory study has demonstrated the value of investing further research to test and to develop this measure. In particular future studies should aim to replicate these findings in more diverse populations and to assess the scale’s longitudinal consistency and sensitivity to change in intervention contexts.

## Data Availability

Data are available from the corresponding author upon reasonable request.

## Appendix A: Escala de Orientação Mística Revisada

Mystical Orientation Scale Revised (MOSR)

As seguintes afirmações tratam de experiências especiais. Indique, por favor, o quanto cada uma das experiências é importante para a **sua** vida, circulando um número entre 1 e 5.

These statements are to do with special experiences. Please indicate how importante each experience is to your life by circling a number between 1 and 5. 1 = pouca importância (low importance); 3 = média importância (medium importance), 5 = alta importância (high importance)

**Table:**

1. Experimentar algo que eu não conseguiria descrever com palavras	Pouca	1	2	3	4	5	Alta
*experiencing something I could not put into words*	*low*						*high*
2. Me sentir tocado(a) por uma força indescritível ……………………………	Pouca	1	2	3	4	5	Alta
*feeling moved by a power beyond description*	*low*						*high*
3. Estar consciente de coisas que eu jamais poderia descrever …………	Pouca	1	2	3	4	5	Alta
*being aware of more than I could ever describe*	*low*						*high*
4. Perceber um sentido na beleza da natureza ……………………………….	Pouca	1	2	3	4	5	Alta
*sensing meaning in the beauty of nature*	*low*						*high*
5. Saber que eu estou envolvido(a) por uma presença …………………………	Pouca	1	2	3	4	5	Alta
*knowing I was surrounded by a presence*	*low*						*high*
6. Ouvir uma voz interior falar comigo …………………………………………	Pouca	1	2	3	4	5	Alta
*hearing an inner voice speak to me*	*low*						*high*
7. Enxergar a essência das coisas por breves momentos …………………	Pouca	1	2	3	4	5	Alta
*seeing brief glimpses into the heart of things*	*low*						*high*
8. Ter inspirações momentâneas sobre o que realmente importa ………	Pouca	1	2	3	4	5	Alta
*having flashes of insight into what really matters*	*low*						*high*
9. Experimentar momentos passageiros de profunda inspiração interior	Pouca	1	2	3	4	5	Alta
*experiencing passing moments of deep insight*	*low*						*high*
10. Ser tomado por uma sensação de estar maravilhado(a) …………………	Pouca	1	2	3	4	5	Alta
*being overwhelmed by a sense of wonder*	*low*						*high*
11. Estar fora do meu corpo de uma maneira misteriosa …………………	Pouca	1	2	3	4	5	Alta
*being in a state of mystery outside my body*	*low*						*high*
12. Ser tomado(a) por um poder além do meu controle ………………………	Pouca	1	2	3	4	5	Alta
*being grasped by a power beyond my control*	*low*						*high*
13. Me sentir Um* com o universo ………………………………………………	Pouca	1	2	3	4	5	Alta
*feeling at one with the universe*	*low*						*high*
14. Ser absorvido(a) por uma força ou Ser maior. ………………………………	Pouca	1	2	3	4	5	Alta
*being absorbed within a greater being*	*low*						*high*
15. Sentir a junção do passado, presente e futuro ………………………….	Pouca	1	2	3	4	5	Alta
*sensing the merging of past, present and future*	*low*						*high*
16. Me sentir Um* com todos os seres vivos …………………………………	Pouca	1	2	3	4	5	Alta
*feeling at one with all living beings*	*low*						*high*
17. Me perder em um Ser maior ………………………………………………….	Pouca	1	2	3	4	5	Alta
*losing my everyday self in a greater being*	*low*						*high*
18. Estar consciente da atemporalidade e da eternidade …………………	Pouca	1	2	3	4	5	Alta
*being conscious only of timelessness and eternity*	*low*						*high*
19. Me sentir mergulhado(a) nas profundezas da existência ……………….	Pouca	1	2	3	4	5	Alta
*feeling my everyday self-absorbed in the depths of being*	*low*						*high*
20. Perder a noção do tempo, lugar e pessoa …………………………………	Pouca	1	2	3	4	5	Alta
*losing a sense of time, place and person*	*low*						*high*
21. Sentir a unidade em todas as coisas ……………………………………….	Pouca	1	2	3	4	5	Alta
*sensing the unity in all things*	*low*						*high*

*Um – no sentido de unidade. **One – in the sense of unity.*

Permissão: A *Escala de Orientação Mística Revisada* é livre para uso, desde que citada a fonte.

Permission: The Revised Mystical Orientation Scale is free to use, provided the source is cited.
